# Do work relationships matter? Characteristics of workplace interactions that enhance or detract from employee perceptions of well-being and health behaviors

**DOI:** 10.1080/21642850.2014.933343

**Published:** 2014-08-11

**Authors:** Karen Mastroianni, Julia Storberg-Walker

**Affiliations:** ^a^ Dimensions in Occupational Health and Safety, Inc., Raleigh, NC, USA; ^b^ Workforce and Human Resource Education Program, NC State University, Raleigh, NC, USA

**Keywords:** occupational safety and health, sleep disorder, quality of life, mixed-methods research

## Abstract

This qualitative case study adopted the position that health and health behaviors are complex social constructs influenced by multiple factors. Framed by the social ecological model, the study explored how work interactions enhanced or detracted from the perceptions of well-being and health behaviors. Despite the fact that previous studies indicated that the social workplace environment contributed to employee health, there was little information regarding the characteristics. Specifically, little was known about how employees perceived the connections between workplace interactions and health, or how social interactions enhanced or detracted from well-being and health behaviors. The participants included 19 volunteers recruited from four companies, who shared their experiences of workplace interactions through interviews and journaling assignments. The findings indicated that feelings of well-being were enhanced by work interactions, which were trusting, collaborative, and positive, as well as when participants felt valued and respected. The study also found that interactions detracted from well-being and health behaviors when interactions lacked the aforementioned characteristics, and also included lack of justice and empathy. The enhancing and detracting relationships generated physical symptoms, and influenced sleeping and eating patterns, socializing, exercise, personal relations, careers, and energy. Surprisingly, the study found that regardless of how broadly participants defined health, when they were asked to rate their health, participants uniformly rated theirs on physical attributes alone. The exclusive consideration of physical attributes suggests that participants may have unconsciously adopted the typical western medical view of health – an individually determined and physiologic characteristic. Despite research suggesting health is more than biology, and despite defining health broadly, participants uniformly adopted this traditional view. The study also offers human resource development professionals with evidence supporting interventions aimed at minimizing workplace incivility. Interventions designed to improve employee engagement could minimize financial and human costs of negative interactions. The bottom line is that workplaces should be physically, emotionally, and psychologically safe for well-being and healthy behaviors to flourish.

It's your values, your core values that get compromised. What you believe in … She said there was a difference between self-confidence and self-assurance. She said that self-confidence is being comfortable with what you know; your expertise on a subject. And that self-assurance is who you are. Lindsey went on to exclaim that interactions that blame or accuse, and lack respect and trust, target self-assurance. She slowly and with emphasis said that, ‘ … who I am, is being questioned and attacked and that's what is so bad to deal with’. (Lindsey, research participant, Mastroianni, 2012)

Based on this exploratory qualitative case study, the answer to the question, ‘do work relationships matter?’ is a resounding yes. The above quote was one of many shared by participants regarding how work interactions matter. This study adopted the position that health and health behaviors are complex social constructs influenced by multiple factors. Framed by the social ecological and communities of practice (CoP) models, the study explored how workplace interactions enhanced or detracted from perceptions of well-being and health behaviors.

Too often workplace health-promotion interventions target individual employees with little or no consideration of other determining factors that impact health and health behaviors (Best et al., 2003; Cherniack, 2013; Keyes & Grzywacz, 2005; Maibach, Abroms, & Marosits, 2007; Stokols, 1992). The interventions are primarily focused on physical health such as weight, fitness, and biometric measures (Aldana, Merrill, Price, Hardy, & Hager, 2005; Anderson et al., 2009; Baker et al., 2008; CDC, 2009; Goetzel, 2011; Goetzel, Ozminkowski, Pelletier, Metz, & Chapman, 2007; Merrill, Hyatt, Aldana, & Kinnersley, 2011; Naydeck, Pearson, Ozminkowski, Day, & Goetzel, 2008; Ozminkowski et al., 2004; K. A. Pelletier, 2009). The effectiveness of such initiatives, including the return on investment (ROI), has been called into question (Emerick & Lewis, 2013; Lewis, 2012; Terry, 2012) and this concern is not new (Schneider & Stokols, 2009; Syme, 2003). In fact, many in the field are emphasizing the need for worksite wellness programs and occupational health to move beyond a health risk/health cost-savings model toward one of productivity and enhancement (Goetzel & Ozminkowski, 2008; Goetzel, Schoenman, Chapman, Ozminkowski, & Lindsay, 2011; Keyes & Grzywacz, 2005; McCunney, 2001; Terry, 2012).

Several large and well-funded studies indicated that individual behavior change methods alone have not been effective in community wellness initiatives either (Serxner, Gold, Meraz, & Gray, 2009; Syme, 2003). For example, in the Multiple Risk Factor Intervention Trial (MrFit), the results showed no difference in heart disease rates between the research participants and the control group (Syme, 2003). What is even more striking is not just that a risk reduction had not been found, but that most participants reverted back to previous habits prior to completing sessions on the healthy behaviors (Schneider & Stokols, 2009). Commit was another well-funded study on smoking cessation interventions. The results indicated that participant smoking quit rates were no different than the general population (Syme, 2003).

One of the chief researchers, Dr. Syme, who was involved in both of these large and expensive individual change initiatives, became an outspoken advocate for addressing broad social and environmental factors rather than focusing only on individual behavior change (Syme, 2003). The bottom line is that individual behavior change methods alone have not been effective in communities nor, in fact, in workplace health-promotion programs.

An independent Task Force on the Guide to Community Preventive Services, with support from the CDC and US Department of Health and Human Services, completed a comprehensive and systematic literature review in 2007. The review focused on the health and financial impacts of workplace health-promotion services targeting obesity. More than 50 companies met the research and program criteria, with the costs for the weight loss interventions ranging from approximately $8500 to $75,750 (Serxner et al., 2009). Yet with few exceptions, the average weight loss was only 3 pounds during 6–12-month follow-up evaluations (Serxner et al., 2009). This does not necessarily mean that there was a program failure, as weight control and preventing unsustainable weight loss are both positive outcomes. In a recent presentation, Edington (2014) emphasized that the goals should be keeping individuals from getting worse and keeping the risk low.

As part of this, many worksite wellness programs focus on health risk assessments (HRAs) and biometric screenings to identify and control risks. However, Soler et al.’s (2010) research regarding the effectiveness of HRAs, as well as HRAs with follow-up, had similar dismal findings. Many of the companies presented in their review were in favor of HRAs, yet only small or modest gains were found and several potential sources of bias were noted. Still, there were more meaningful outcomes for reducing tobacco use, alcohol use, dietary fat intake, blood pressure, cholesterol, and increasing seatbelt use when follow-up was conducted after HRAs. However, the outcomes were based on unsubstantiated self-reporting. Regarding nutrition and weight loss, the findings were dismal due to the intake of fruits and vegetables, body composition, and physical fitness. O'Donnell (2013) stated that the findings were not surprising based on his research, confirming that HRAs and education alone were not enough. He also noted that, ‘ … it is important to acknowledge that we have not been successful in developing programs that are predictably successful in helping people lose weight, increase fitness level, or increase their fruit and vegetable consumption’ (O'Donnell, 2013, p. iv). Actually, the only programs with the most positive ROI had low per person annual costs (Cherniack, 2013) or were larger, more comprehensive approaches to wellness such as programs at Citibank, Johnson & Johnson, and Proctor and Gamble (Goetzel, 2013). Again, indicating what researchers acknowledge and what has been slower in practice, much broader and multilevel approaches are needed for sustainable health behavior changes.

This being said, positive workplace wellness ROI studies have been published related to absenteeism and disease management (Aldana et al., 2005; Baicker, Cutler, & Song, 2010; Chapman, 2012; Pellitier, 2009; Serxner, Alberti, & Weinberger, 2012). A systematic review and analysis of published workplace health-promotion research indicated that positive clinical and cost-saving outcomes were mostly due to disease management programs (Chapman, 2012; Pellitier, 2009; Serxner et al., 2012). A quasi-experimental study investigating the effectiveness of health and productivity management (HPM) found a cost-saving ROI during years 2 and 3, but with a slighter decrease (Serxner et al., 2012). The HPM consisted of HRAs, screenings, and disease management for those with high risk factors.

Disease management has become the current trend for healthcare cost-saving initiatives in the workplace and is also described as workplace wellness programs (Goetzel & Ozminkowski, 2008; Goetzel, Ozminkowski, et al., 2007; Goetzel, Shechter, et al., 2007). The cost–benefit results from the focus on high-risk individuals who had higher medical costs and were absent more from work (K. A. Pelletier, 2009; Serxner et al., 2012). The outcomes were reduced absenteeism as well as fewer hospitalizations and doctor visits.

Other studies found no significant differences in healthcare costs between those who participated in any one wellness program during a year time-frame with those who did not participate (Aldana et al., 2005). The findings, however, again indicated a significant negative association between participation and absenteeism, with nonparticipants having a higher rate of absenteeism than employees who participated in any one program during the one-year period. A recent meta-evaluation on worksite health-promotion ROI found that despite many design and measurement inconsistencies, there were strong reductions in sick leave, health plan costs, workers' compensation, and disability insurance (Chapman, 2012). One meta-analysis mentioned was by Baicker, Cuttler and Song. The researchers found an ROI of $3.27 for medical cost savings and $2.73 for absenteeism reduction based on 22 studies examined. However, Dee Edington (Terry, 2012) stated that he had not found ‘an accurate and reliable method to get a true measure of ROI’ (p. 4), even calling some research summaries a reckless use of ROIs. Instead, he recommended the use of cost trends.

## Health as a complex concept

What the research in this area lacks is consideration for the complexity of health and health behaviors beyond a focus on disease. Another concern is that combining disease management with wellness initiatives makes it difficult to determine the effectiveness. In addition, this attention perpetuates a disease mentality that focuses on individual medical conditions and physical health risks alone. Literature supports that sustained health behaviors are not accomplished without consideration of other determining factors, including other dimensions of health in addition to the physical as well as social determinants of health. There are the models of other health dimensions besides physical health, such as Five Dimensions by O'Donnell (1989) that includes physical, spiritual, emotional, social, occupation, and intellectual; Hettler's (1976) Six Dimensions of Health that includes occupation; and Rath and Harter (2010) dimensions of career, physical, financial, community, and social. These dimensions are rarely ever considered in most workplace wellness programs, and when they are considered it's without any level of significance. Keyes and Grzywacz (2005) emphasize that not doing so cannot lead to complete health. Their research indicates that complete health balances the absence of morbidity with the presence of well-being.

A previous study analysis revealed that few of the wellness interventions addressed the physical, psychosocial, or corporate policy impact on employee health (Keyes & Grzywacz, 2005; K. R. Pelletier, 2001). In 2001, K. R. Pelletier (2001) cited the importance of the impact of corporate culture, including supervisor and co-worker support for improved health status. The author noted:

Few of the interventions cited here focused on the physical, psychological, or policy work environment and its role in employee health … it is evident that employees need to know that their organization is seriously concerned about their health … Employees need to perceive that their senior management, supervisors, and co-workers have positive attitudes toward health since these factors have all been associated with improved employee health status. Interventions and evaluations of workplace programs may benefit from including such components and measures of the work environment in order to determine the influence of such factors on the overall clinical effectiveness and cost-effectiveness of these interventions. (K. R. Pelletier, 2001, p. 115)

Research demonstrates that organizational social and physical environments exert considerable influence over the choices that individuals make, the resources available to make those choices, and the factors that influence health status (Green & Kreuter, 2005; Institute of Medicine, 2001; Schneider & Stokols, 2009). Simply focusing on individual responsibility for health and adopting healthier behaviors ignore the influence of contextual factors that shape behaviors (Cherniack, 2013; Goetzel et al., 2007; O'Donnell, 2013; K. R. Pelletier, 2001; Peterson & Wilson, 1998; Stokols, Pelletier, & Fielding, 1996). Participation has been low, the results have been less effective, and have also sent mixed messages about the importance of practicing healthier behaviors (Crump, Earp, Kozma, & Hertz-Picciotto, 1996; Linnan, Sorensen, Colditz, Klar, & Emmons, 2001; Linnan, Weiner, Graham, & Emmons, 2007; MacDermid et al., 2008). Dee Edington (Terry, 2012) captured this sentiment when he stated:

Behavior change is really the mantra of wellness, but if a person achieves a lifestyle behavior change, only to return to the same unhealthy environment, what can we expect will happen? We set up wellness for failure if we don't work on improving the environment & culture before we work on individual behavior change. (TAHP, p. 5)

Current research on motivation and well-being adds additional elements to consider (Deci & Ryan; Pink, 2009). Deci and Ryan's self-determination theory (SDT) suggests that humans have a basic need for autonomy, competence, and relatedness, and also tendencies toward growing, mastering challenges, and integrating new experiences (with support and barriers). Essential to the theory is the workplace in terms of these supports and barriers. SDT propositions focus on how social and cultural factors facilitate or undermine people's sense of volition and initiative, in addition to their well-being and the quality of their performance (http://www.selfdeterminationtheory.org/theory/, last accessed June 6, 2014). Similarly, Pink (2009) found that employees desire autonomy, mastery, and meaning over money or other extrinsic rewards. At this level, wellness programs move beyond health risks, individual focus and healthcare cost savings, to create thriving and prosperity for individuals and organizations. Deci (1995) noted that there are hundreds of scientific studies which have demonstrated that autonomous behavior results in more creativity, problem-solving, more positive emotions and better physical and psychological health.

The review of this body of knowledge on health-promotion interventions implies that it is necessary to develop multilevel organizational interventions rather than focus exclusively on individual employee health behavior (Kok, Gottlieb, Commers, & Smerecnik, 2008; Schneider & Stokols, 2009). This is not to argue that skills, knowledge, and intention are not necessary components for behavior change, but their influence on the physical and social environment as barriers or supporters of the behavior cannot be denied (Blunt & Hallam, 2010; Crosby, Kegler, & DiClemente, 2009; Green & Kreuter, 2005). By doing so, places the responsibility solely on individual employees, virtually ignoring the impact that the working environment may have on health and unhealthy behaviors.

In fact, studies suggest that negative interpersonal interactions, such as mistrust, hassles, and criticisms, have detrimental health consequences (Heaney & Israel, 2008; Pearson & Porath, 2009). Of particular interest is the fact that incidents of myocardial events occur more often on Mondays (Mackey & Sisodia, 2013). The authors stated, ‘The workplace is frequently a pressure-cooker environment, working conditions are often poor, team members are not valued and colleagues view one another as competitors and threats’ (p. 85). Pearson and Porath (2009) researched the prevalence of these uncivil behaviors for the past decade. The authors' findings illustrate the consequences for both the employees as well as the organization in terms of stress, lost productivity, and even sabotaging behaviors (Pearson & Porath, 2009). These social interactions, or work relationships, have been identified as key elements in healthy workplace models, and yet the social component of work organization has received little attention when designing health-promotion programs (DeJoy & Wilson, 2003; MacDermid et al., 2008; MacIntosh, MacLean, & Burns, 2007; Quick, Macik-Frey, & Cooper, 2007; Wilson, DeJoy, Vandenberg, Richardson, & McGrath, 2004). Several researchers advise that more knowledge is needed regarding the social aspects of the work environment and potential impact on health behavior, as well as, the impact social aspects also have on organizational performance (Golaszewski, Allen, & Edington, 2008; Linnan et al., 2007; Lowe, Schellenberg, & Shannon, 2003; Wilson et al., 2004). In fact, when testing their model of healthy organizations, Wilson et al. (2004) noted that

The social domain of work is probably the most intriguing and the least understood of the constructs studied and yet has a major influence on the efficiency and effectiveness of the organization. (p. 582)

While organizations are beginning to address the physical environment (Blunt & Hallam, 2010; Chu & Dwyer, 2002; Goetzel, Ozminkowski, et al., 2007), the social environment has not been effectively studied to date (Blunt & Hallam, 2010; Wilson et al., 2004). Studies have demonstrated that supportive changes in the physical environment, such as healthy options in vending machines and cafeteria, walking trails, exercise facilities and flexible work schedules, positively influence health behavior, but the social environment also matters (Blunt & Hallam, 2010; Crump et al., 1996; Linnan et al., 2001; Wilson et al., 2004). Conclusions from their study on physical environmental changes to support exercise, Blunt and Hallam (2010) acknowledged that organizational social support is important to individuals' perception of the environment and the need for future research to address this aspect. Crosby et al. (2009) also noted the social influences of health and health risks behaviors, and stated that ‘The profound role of social determinants in shaping health behavior is becoming increasingly apparent’ (Crosby et al., 2009, p. 6).

### Determining factors for health

From this perspective health is not just a biophysical condition, but a dynamic process that includes a social component. Health and health behaviors are entwined with well-being and grounded within the social context of living. The United Nations Educational, Scientific and Cultural Organization (UNESCO) defined health as, ‘ … essentially a social construct: it is created in the interaction between people and their environments in the process of everyday life: where people live, love, learn, work, and play’ (Daley, Merriam, Courtenay, & Cervero, 2006, p. 232). Health cannot be broken down into components, but must be understood from this comprehensive and holistic definition. Well-being is no longer separate from health, but from this definition is the overarching construct, with health as a subset of well-being (Danna & Griffin, 1999).

Using a social ecological framework instead of individual behavior change models supports this understanding. In addition to individual change, addressing the physical and the social workplace environment needs to be considered for sustainable health practices. With this consideration, workplace health promotion cannot be effective by focusing on individual risk factors alone, such as reducing blood pressure or cholesterol levels, increasing physical activity, or reducing obesity. Instead, the broader components need to be addressed, including the physical and social issues of the workplace that may influence health and health behaviors.

This broader focus is not to deny individual responsibility, but only to establish the need to consider additional influential factors. The need for behavior change and health-promotion education interventions is to assist individuals in smoking cessation, healthy eating, weight loss, increasing exercise, and controlling cholesterol, blood pressure and blood sugar levels (Glanz, Rimer, & Viswanath, 2008; Mokdad, Marks, Stroup, & Gerberding, 2004); however, the solutions are not easy, and addressing the health risks is far more complex than targeting individual behavior change alone. What is striking is that behavior change initiatives are primarily focused on a medical disease model of finding and treating pathogenic conditions. This attempts to reduce health to an absence of disease without regard to overall well-being and determining factors that influence health behaviors. Yet, much of adult lifestyle habits are culturally ingrained and reinforced by our work and personal environments, special interest groups, and environmental barriers and cues (DiClemente, Crosby, & Kegler, 2009). Although education remains important, health education is not a monolithic activity (DiClemente et al., 2009, p. 565), but requires attention to various determinants of health and behavior.

This scope moves beyond the definitions currently popular in worksite wellness programs. The historical definition of health is the absence of disease (Danna & Griffin, 1999). The World Health Organization expands this definition and begins to connect health and well-being by adding that ‘health is not only the absence of infirmity and disease but also a state of physical, mental and social well-being’. The best known definition in the profession of health promotion – which has remained championed from its inception – is that health is an optimal state or balance of physical, emotional, social, spiritual and intellectual health (O'Donnell, 1989, 2002, 2009). These definitions imply that health is more than just being disease-free; however, the definitions also indicate health as a state or condition, without consideration for the dynamic and complex aspects of health. Also, as stated earlier in this article, it is the physical dimension of health that garners the most emphasis.

The broader definition of health shifts the focus from a medical model and disease risk factors to intersect with well-being. In the past, well-being has been associated with mental health, but is more broadly defined as people's positive evaluation or perception of their lives, including positive emotions, engagements, satisfaction, and meaning (Allen, Carlson, & Ham, 2007). Actually, the perception of well-being and its impact on medical or physical health has been demonstrated to be a strong indicator of actual health outcomes (DeSalvo, Bloser, Reynolds, Jiang, & Muntner, 2006; DeSalvo, Fan, McDonell, & Fihn, 2005). In their studies, questions such as rating ones health as poor, fair, good, or excellent, and rating one's health in comparison to others with similar demographics were more predictive of health status than objective health measures such as blood pressure, blood cholesterol, exercise, or even weight.

In this current research, the concept of well-being is seen as the broader and more encompassing construct, with health being a subset of well-being (Danna & Griffin, 1999). Ryff and Singer (1998) provided a concise summary of well-being as an umbrella term, which encompasses the UNESCO's definition and describes health and well-being using three principles:

- Health is not a medical question but a philosophical position pertaining to the meaning of the good life for each individual.
- Health includes the interconnection of mind and body.
- Health is multidimensional and dynamic process; that is an engagement in living and is an expression of human potential of intellect, social, emotional, and physical.

From this broader perspective, well-being and health are used interchangeably. This runs parallel with the current emphasis in the field of psychology on helping individuals thrive and flourish. This is a change from a disease and risk-based approach, an important and interesting note since the wellness behavior change models are based on psychology and medical models. Termed as Positive Psychology, the approach considers how three critical dimensions of life experience – pleasure, engagement and meaning – combine to create life satisfaction. It is a movement toward fostering what makes people feel happier and more resilient rather than treating mental illness (Seligman, Steen, Park, & Peterson, 2005).

In the discipline of positive psychology, the term ‘character strength’ is used to understand how people interact with each other. It is reasonable to suggest that the characteristics of workplace relationships found in this current study and the character strengths found in the positive psychology literature are connected in some way. For example, 24 character strengths have been identified that provide benefit to health, relationships, and careers (Seligman & Csikszentmihalyi, 2000). The empirical findings show that the character strengths are valued by adults around the world, including all states within the USA. The most commonly endorsed strengths are kindness, fairness, authenticity, gratitude, and open-mindedness, and the lesser strengths consistently included prudence, modesty, and self-regulation. It is not a stretch to realize the impact of these strengths on health and well-being.

Surprisingly, the complexity and social aspects of health as well as health behaviors, leading to sustainable healthy lifestyle changes were originally the intent of the *Surgeon General* Report (U.S. Department of Health, Education, and Welfare, 1979). A social ecological framework provides the justification for exploring the influence of interpersonal determining factors on health and health behaviors within workplace interactions.

## Study purpose

Therefore, the purpose of this exploratory qualitative study was to understand how employees perceived their workplace interactions as helping or hindering their perception of well-being, and how these interactions influenced health behavior practices (Mastroianni, 2012). This is just one slice of determining factors that may influence health behaviors. Qualitative inquiry was the selected research methodology since it provided the methods to examine social phenomena and individual relationships within their naturally occurring environments. Qualitative research affords a comprehensive approach to study social phenomenon based on a constructivist or critical perspective, and provides a variety of approaches to select from according to the intent of the study (Bloomberg & Volpe, 2008).

## Conceptual frameworks

Two conceptual frameworks support this approach. First, from the discipline of public health (Green, Richard, & Potvin, 1996; Maibach et al., 2007; McLeroy, Bibeau, Steckler, & Glanz, 1988), the ‘social ecology model’ provides the justification for expanding the scope of health and wellness research beyond the individual level of analysis. In other words, this accepted framework for understanding health suggests that health is a multidimensional construct involving the individual person AND the social and physical environments surrounding the person. The second conceptual framework is referred to as ‘CoP’ from the field of anthropology (Lave & Wenger, 1991). This model is ontologically aligned with the social ecology model, and is used in this study to analyze employee work relationships.

### Social ecology model

As previously mentioned, a social ecology model considers the determining factors on health and health behaviors, allowing for methods to address not just individual risk factors, but the social and contextual influences on both (Stokols, 1996). These factors include intrapersonal (individual characteristics, skills, behaviors, and health risks); interpersonal (social influences at home and work); organizational/institutional factors (physical and social environment, policies and procedures, and work processes); community factors (relationship and boundary between organizations); and public policy (local, state, and national laws and policies) (McLeroy et al., 1988). Studies have demonstrated the influence these determining factors, especially the interpersonal factors, have had on making and sustaining healthy changes such as increasing physical activity (Payne, Jones, & Harris, 2002; Trost, Owen, Bauman, Sallis, & Brown, 2002), participation in onsite programs (Crump et al., 1996; Linnan et al., 2007; Sloan & Gruman, 1988), and perceptions of well-being and quality of life (Lowe et al., 2003; MacDermid et al., 2008). In fact, DeJoy and Wilson (2003) coined the term *organizational health promotion* to expand the concept of workplace health-promotion programs beyond a focus on individual employees. This supports the impact that organizational structure, work operations, and social climate have on employee health and performance.

Social ecology provides an integrative framework, that is, a comprehensive approach to address these determining factors of health and health behavior. This is not to discount individual responsibility for health and healthy lifestyle choices, but only to acknowledge the complexity of health and healthy behaviors. Chronic diseases, causes of death, disability, and lifestyle behaviors are complex and influenced by a variety of factors (Gielen, McDonald, Gary, & Bone, 2008; Glanz & Rimer, 2008; Rath & Harter, 2010; Schneider & Stokols, 2009). The ecological model is based on the premise that health is a product of interdependence between individuals and several sociocultural systems (Green et al., 1996). When viewing health and health behavior as complex systems within an ecological framework, providing individuals with motivation and skills to change behavior cannot be effective if environments and policies make it difficult or impossible to choose healthful behaviors.

### Communities of practice

One way to understand the social interactions in organizations is through the lens of CoP. The CoP components include meaning, practice, community, and identity (Wenger, 1998). It is not merely considering each separate component, but the intersection or interconnectedness between each component that forms a CoP. It is the action, sense of belonging and participating in the CoP that not only shapes what individuals do, but who they are, how they interpret what they do, and who they become (Lave, 1991; Storberg-Walker, 2008; Wenger, 1998).

The CoP framework helps to understand learning, meaning making, identity formation, and participation. It is through participation – social interactions – that members of a community learn and form their identities, and develop shared meanings. Other studies using CoP as the theoretical framework for health behaviors have not yet been found; however, the model was used to explain workplace safety behaviors (Gherardi & Nicolini, 2000; Machles, 2004; Machles, Bonkemeyer, & McMichael, 2010). For example, when researching safety training for a construction company, Gherardi and Nicolini (2000) concluded that the abstract classroom safety training was assimilated according to the culture of the CoP and would thereby discount the training. Similarly, in his dissertation, Machles's (2004) findings indicated that social learning was significant within workgroups and was recognized by the participants in his study as the most reliable and accurate information compared to other workplace training. The participants learned safe practices through the interaction with co-workers. Another study illustrated that utilizing the knowledge within the CoP of field engineers resulted in better solutions, reduced injuries, lowered workers' compensation costs, and improved communication (Machles et al., 2010).

Because of this combination of social learning and identity formation, CoP is selected as the theoretical framework to explore the characteristics of workplace relationships, and how perceptions of health and health behaviors may be influenced by these social interactions. Based on the idea that health and health behaviors are complex concepts that are socially defined and directly influenced by the organizational environment, it is natural progression to consider that social interactions within the workplace influence health practices and perceptions of well-being.

Grounded on the CoP lens and the broad and social definition of health used for this research, an overview of results are summarized below.

## Research findings

Approval from the NC State University Institutional Review Board was obtained to ensure that the highest standard of research ethics was upheld. The research participants included 19 volunteers recruited from 4 companies, who shared their experiences and perceptions of workplace interactions through two interviews and journaling to capture real-time interactions. The participants' discussions and journals provided rich stories that were coded and classified as characteristics that influenced well-being. Data analysis included open coding to identify themes of the interaction characteristics, followed by a second phase of analysis to code CoP key elements of meaning, learning, and identity. Experts in the field reviewed and confirmed the codes and characteristics identified.

The participants' backgrounds, education, work experiences, and job positions varied. Of the 19 participants, 14 were females and 5 were males. The age range was between 34 and 57 years, and the participants had been with the perspective companies from 1 year up to 20 years. Education ranged from high school degrees to doctorates. Participants were ethnically diverse as well, with 12 self-identifying as Caucasian, two as Asian, one person as Sri Lankan, one as Indian-American (parents were originally citizens of India), one as African-American, one as black, and one as American (one parent was Italian and one African-American).

The participating companies also varied in terms of types of business, as well as cultures; however, all were recruited based on their health-promotion programs. The types of businesses included pharmaceutical, biotechnology, agriculture, and personal care product manufacturing. The company sizes ranged from 250 to 850 employees. Two of the companies had multiple locations and the research volunteers were from both of the company facilities. Three of the companies had been recognized and received rewards for their health-promotion programs and one had a strong commitment to employee health and safety. This information lends strength to the suggested findings, since regardless of the differences and the different stories shared, the emerging characteristics were strikingly similar.

The study found that the perceptions of well-being and practicing healthy behaviors were enhanced by perceptions of work interactions that were trusting, collaborative, open, positive, and social, as well as when participants felt valued and respected. The study also found that work interactions could detract from perceptions of wellness and well-being. These detracting types of interactions lacked the aforementioned characteristics, and also included relationships that were perceived to be lacking justice and empathy. The enhancing and detracting interactions generated physical symptoms, as well as influenced the sleeping and eating patterns, socializing, physical activity, emotional well-being, personal relations, career decisions, and energy levels. Tables 1 and 2 summarize the characteristics that emerged from the data. Three professionals in the field validated the emerging characteristics from the data.

**Table 1.** Summary of characteristics that enhance or detract from well-being.

Characteristics that enhanced well-being	Characteristics that detracted from well-being
Collaboration/teamwork	Lack of collaboration/teamwork
Mutual respect	Disrespect/condescending
Trust	Lack of integrity/distrust
Open, clear communication	Lack of open communication
Valued/recognition	Lack of value/recognition
Socializing/personal connection	Difficult interactions
	Injustice/lack of fairness
	Lack of empathy

**Table 2.** Summary of influence on well-being and health behaviors.

Categories of well-being and health behaviors influenced
Physical symptoms
Sleep
Eating
Exercise
Energy level
Emotional health
Social impact
Personal relationships
Career impact

Data analysis using the CoP framework found that meaning, learning, and identity emerged when participants discussed detracting interactions with colleagues. These discussions helped to minimize the influences on well-being and health behaviors. In fact, the only participant who maintained weight loss through company-sponsored wellness activities described continuing weekly support meetings with co-workers to encourage healthy eating and exercise. They had formed a CoP to support each other's weight loss. The other participants reported gaining all or more weight following a wellness initiative.

Participants also made sense of detracting interactions by discussing the situation with co-workers or other friends. They learned a different perspective or confirmation of their perception, which kept their work identities intact. They expressed that this enhanced their well-being and when this support was not available, it had a detrimental effect on perceptions of well-being and health practices as well as on their professional identities.

Below are a few highlights of stories shared by participants.

## Shared stories

Health behaviors and well-being were influenced by work interactions, regardless of other supporting wellness programs. Work interactions influenced energy levels while working, as well as when at home, and influenced healthy behaviors. Participants' expressed having less tolerance, patience, and/or willingness to complete normal work activities, as well as activities in their personal life. For example, Lois expressed, ‘I'm more tolerant at home on the good days than I am on the (pause), because, you know, sometimes when I'm not feeling good about myself then I have a short temper, other people (at home) suffer, unfortunately.’ Enhancing workplace interactions had the opposite influence on energy levels. For example, Chelsea excitedly described that when she felt listened to, respected, and given flexibility to do her work, she would, ‘go the extra mile’. The health impact was, ‘it makes me feel energized, it makes me feel awesome, it makes me feel like I have a worth!’

The workplace interactions also had an effect on sleeping and eating patterns. When dealing with detracting interactions, Bridgett described sleeping poorly for days, and then her health, physical activity, as well as her work productivity suffered. She described not feeling well and not being able to concentrate. One story Bridgett shared was when she made a cynical remark to her manager regarding another employee who Bridgett described as ‘a problem employee’. Bridgett's response to her manager was that it ‘wasn't her day to watch her’, when the manager asked her where the employee was. She said her boss ‘conked’ her in the head for saying it was not her day to watch the employee. Bridgett recalled, ‘And she hit me in the head. She didn't just tap me; she hit me hard in the head.’ When probed about the story Bridgett said that she felt she deserved being hit, and though she admitted now that something was wrong about that incident, she could not articulate it fully. She only said that she did not really understand ‘what the hell I had done’. She did not report the manager to Human Resources (HR) because she did not perceive that HR would assist. This was a perception shared by most of the study participants. They would not go to HR or if they had, they felt it was not beneficial and actually harmful.

Example after example of these experiences was shared during the interviews or in the journals. Several shared stories of interactions with their managers or a co-worker that were uncivil, including yelling, being verbally abusive, or slamming their fists on a desk. Madison discussed an interaction that she described as a misunderstanding with a manager that was taken out of context. She said the interaction became ‘heated’ and he raised his voice. The harsh interaction led to his inability to hear her explanation. Madison was shocked and her well-being was influenced by ‘being grown and being yelled at’.

(Nervous Laughing) Yeah, I wasn't used to that, at all. I was shocked. I think I stood there in shock initially. And I'm like OK, because it took me a minute. Okay, I'm like he's really hollering. He's hollering, he's raising his voice and I'm grown! (laughing) … . I do remember feeling hurt. And then, um, I remember gathering myself together mentally long enough to try to relay to him what *really* [emphasized] happened. You know? What my intents were when I did xyz. But when a person is an employee … and he was yelling (pause). He was not hearing. I don't think he could; to this day he never heard me.

When well-being was enhanced – participants felt valued, respected, listened to, trusted and appreciated – they described feeling that employees had each ‘other's backs’, they enjoyed coming to work, and as one described, ‘it was like jazz’ with everyone's contributions being considered and worthwhile. As Madison said,

 … when you feel good and you feel like you have a zest for life that naturally makes you feel like you have a little bit more energy so you come home and you like, you know, ‘I think I'm going to go and walk around the block,’ you know? Or I might go exercise or take the kids out to the park. Or you *want* [emphasized] to eat healthy. I think it's just positive, brings about more positivity. Where, having a bad day makes you just want to go home and do more bad things, you know?

Also, several participants mentioned that enhancing interactions also had a relaxing and calming effect. Kirk said that when he felt listened to and valued, he experienced a sense of relaxation. He described that his breathing was slower; he felt centered and relaxed; and he could think more coherently. Like Kirk, Scarlett also reported being calmer. Scarlett noted in her journal that a day started calmer and more relaxed when being engaged in positive interactions while working.

Participants discussed a difference in energy levels and rated well-being higher when collaborating with others. For example, Cybil said,

It's the social-slash-collaborating yeah. And working on a common purpose. I think, [it's the] creative spark … I'll bring different people together, having these conversations and having this interaction and we can kind of learn from each other and I like to do that … I get the people together and so we can have these sort of interactions … I mean, we don't always agree, sometimes we have a little bit of friction but it's okay, it's because of respect and trust.

Several described enhanced well-being when feeling that co-workers looked out for each other. Sienna said, ‘… we're watching each others’ backs. It's a safety net. OK, this person has my back because they care about me doing well, I care about them doing well, and we want to make sure that nobody gets any {errors} … ’ Trust, respect and collaboration were components of that sense of caring mentioned by Spock, Scarlett, Thelma, Sienna and others.

On the other hand, when participants did not feel valued, appreciated, respected, or listened to they reported a lack of energy, not eating well, not exercising, and also the contagiousness of the negative feelings. One participant recalled that her manager told her:

‘You're just not worth the money that you make here.’ … I actually said I thought a manager's job was to bring out the best. And he was like, ‘Well, that's not my management style. You have to adapt to me. You have to do what I want.’

She reported not being able to participate in exercise at the gym, the one thing that always helped her feel better. Her sleep was impacted, as well as her eating habits and her interactions with her children.

Similarly, Midge's manager told her that, ‘Well, it doesn't really matter what YOU (*emphasis*) think. It's what **I** [*emphasis*] think at the end of the day that matters, because I'm the manager.’ She described how bad that made her feel and a sense of dread that lasted months. Likewise, Madeline said that her manager told her that the supervisor was always right. She recalled him saying that, ‘Well, she's the boss. You're just an analyst. She's right one-hundred percent of the time.’ She repeated that she was only an analyst, and then went on to explain how contradictory that statement could be, remarking, ‘You're only an analyst, so you don't have any business doing that.’

Detracting interactions had an influence on participant's health. The physical symptoms described included an upset stomach, feeling muscle tension, a sense of dread and actually feeling their blood pressure rise. For example, when a manager took credit for one of the research participant's projects, he recalled:

My shoulders just tighten up and you just feel like your blood pressure and your breathing just sky rockets, and then for about a day of just being angry. But after that your just; that kind of goes away and then you just get, after a while you get apathetic.

Likewise, Spock discussed a sense of dread when he was not able to avoid confrontational co-workers. He stated he felt terrible and described the physical influence as ‘heavy’:

Yeah, I mean, well, either terrible because it went terribly but you had to do it to get things done, or relieved that it wasn't as bad as you played it up in your head. It's going to be one of those two. Either way, there is sort of a heavy feeling in your mouth, throat, and down into your stomach. But yeah, it just sort of sits there.

Spock went on to discuss that it impacted the rest of his day. Lois also mentioned that her day was ruined if she had to interact with a difficult co-worker. For both, they described not being able to concentrate or engage in work activities. Lois described the physical symptoms, as well as the influence the interaction had on her health behaviors. Often venting provided a buffer for Lois, but if there was not a buffer,

I would get physically ill because I would have to deal with a person. And yeah, it ruins your whole day, that's all you can focus on then. And it affects how you deal with the good relationships in your workplace. It affects everything, every aspect of what you're doing. Take it home. That's where I vent. Occasionally I would end up going to bed early or get one of my nasty headaches. You know, stress induced headache.

Like Kirk, Monique described that she could feel her blood pressure rise when in detracting interactions. ‘I can just feel my, you know, getting tense and my blood pressure rising and thinking, “Oh my gosh”. I guess I just feel throbbing and blood rushing to my ears and kind of just, “Ahh”!’ Monique also described that ‘Ahh’, or screaming in her head when dealing with negative complainers. The impact is on physical health. Even though Monique is young and active, with no history of hypertension, she now takes blood pressure medication.

Despite a wellness program with emphasis on preventative health, losing weight, healthy eating and exercise, all participants discussed how work interactions positively (enhancing characteristics) or negatively (detracting characteristics) influenced health behaviors. Madison reported gaining approximately 30 pounds as a result of snacking more while at work. Three participants described this type of mindless snacking as, ‘chocolate therapy’. Several discussed rationalizing while making certain food choices. As Ginger said:

I'm going to go to fast food. I'll rationalize it in my head. I'll justify it in my head. ‘I don't have time for this; I don't have time to cook.’ And these are the inner-dialogues I have with myself, ‘I would love to stop at the store and get myself a nice dinner and cook it, but I am not going home and cooking that, I don't have energy for it.’

She went on to say that on ‘good’ days she was much more likely to make healthier decisions, ‘… I'm much more apt to go home and say, “Hey, we're gonna grill out this evening and I'll stop and get a watermelon.”’

When discussing healthy behaviors, only one of the participants discussed having a schedule for exercising regularly. However, of interest is that others mentioned increasing physical activities only when their companies planned fitness initiatives, but then stopped exercising at the end of the program. One actually stated she hoped there would be another initiative soon since she had gained all the weight lost in the previous initiative. Two others admitted that was the reason they continued as a member of the wellness committee since they were more apt to participate if they helped plan the initiatives. On completion of the research, many anecdotal stories have been shared regarding individuals gaining all of their lost weight or more between wellness initiatives or purposely gained weight to get more of an incentive during the next wellness challenge.

Of interest is that all participants denied that a heavy workload caused distress. It was the interactions to complete the work and sense of or lack of camaraderie that enhanced or detracted from well-being that was stressful or a buffer.

These are just some of the stories shared and examples for several of the characteristics. There are other findings that emerged from the data.

## Additional findings

Other findings included:

1. When core values were impacted, that had the greatest influence on well-being and health behaviors;
2. The ripple effect that both enhancing and detracting workplace relationships had on the contagiousness of the types of interactions, the influence on well-being, and the ripple to relationships inside and outside of work;
3. Nonverbal, as well as verbal workplace relationships influenced health;
4. A sense of compassion for those causing difficult interactions developed;
5. Perceived benefits from the interviews and journals, all described it as counseling sessions;
6. Regardless of how broadly participants defined health and well-being (including spiritual and emotional aspects for example), when they were asked to rate their health, participants uniformly rated on their physical attributes alone; and lastly
7. The perceived lack of support and resources to address detracting characteristics, such as from HR professionals.

A few of these results are worth further discussion. The contagiousness of negative and positive interactions or the ‘ripple effect’ (#2) was mentioned previously. Some described the negativity or toxicity as being consuming. Ginger expressed that she was consumed by the overall negativity at a previous company and resigned because of it.

Oh it was miserable; I can tell you when I worked there … I couldn't stand myself. I hated myself. I mean everything was so negative I got into that negative loop and everything was just a complaint and miserable and just, I was miserable, I hated it. I was absolutely just miserable and you know, my husband said to me, ‘Why are you letting it do that to you? Why are you letting it dictate your state of being and your mind set about things?’ … And I realized that it was the place, I had to leave. There was no way to fix anything and that was extremely frustrating for me and it just consumed me. I mean the negativity just absolutely consumed me, impacting my family and work quality.

One of the interesting findings was #6 – how participants rated their well-being and why. This question was asked in order to generate their understanding of wellness and well-being before beginning the interviews and journaling assignment. Participants were asked to rate their health and discuss a definition of health and well-being. While the data were not connected directly to the research questions, the data were necessary to obtain in order to make sense of the subsequent stories and perceptions relayed by the participants. At the beginning of the study, it was not anticipated that the description and definition of health would offer any findings; however, analysis revealed interesting patterns that were unexpected and meaningful. First, all participants provided a broad and holistic definition of health and well-being, but used a very narrow conception of health when they were asked to describe their own well-being and healthy behaviors. Participants focused only on the physical aspects and did not include social, emotional, or spiritual elements that they included in their definitions. Second, participants generally expressed guilt when talking about practicing healthy behaviors. What they conveyed indicated whatever they did was never good enough to be considered healthy. And third, participants discounted the benefits of their daily physical activities and did not consider such activities as ‘exercise’ or a healthy behavior.

The first finding of interest regarding this is that regardless of their definitions, all of the participants discussed making or needing to make improvements only regarding their physical health when providing a health rating. Participants discussed needing to eat better, exercise more, lose weight, and control biometrics such as blood sugar and blood pressure. None of the participants described having to meditate more, enjoy meaningful work, engage in spiritual activities, or to make more time to laugh or socialize. While the contradiction was surprising – even a participant said that she was surprised – it is certainly aligned with what is emphasized in the workplace health-promotion literature, as well as the media focus.

Much of the literature on worksite health-promotion studies focuses mostly on the physical aspect of individual health risk and behaviors. From this perspective, it is unsurprising that participants would focus on physical health and individual factors alone. However, what is intriguing is that the participants were quickly able to recall story after story about how their workplace interactions influenced their health practices and well-being. Yet they would talk only about physical aspects in this initial realm of inquiry, such as the need to diet, exercise, and so on, but did not make the connection to workplace interactions. Participants seemed to be unaware, on one hand, that workplace interactions were a part of their ‘health’. However, on the other hand, stories were effortlessly shared that described ‘energizing’ or ‘detracting’ interactions at work. None of the participants had fully considered the implications of broader factors of health.

The second finding of interest was a sense of underlying guilt as participants discussed health definitions and ratings, with each sheepishly admitting what they perceived as their unhealthy habits, such as not exercising enough or not making healthier food choices. This was also a finding in a pilot study when participants began discussing what they should and should not be doing regarding health behaviors (unpublished, Mastroianni, 2009). This finding also coincides with similar suggestions of victimizing or blaming the individual that may lead to guilt (Emerick & Lewis, 2013; Golaszewski et al., 2008; Ryff & Singer, 1998). These studies reinforce the status quo or traditional thinking about health and health behaviors – a person ‘ought’ to be able to make the ‘right’ decisions. This narrow perception, as described earlier, does not take into account the broader issues surrounding and influencing health and health behaviors. Consequently, this study suggests that ‘guilt’ is another indicator of a lack of understanding of the complexities surrounding health, and that future health-promotion interventions could be redesigned to focus on workers well-being and all of the various influences on health.

The third finding regarding the definitions was the narrow view participants had of activities that constituted exercise. Participants discounted physical activities during the day, as well as other healthy behaviors, such as lunch choices. This finding was similar to a definition of exercise in the book *Switch* (Heath & Heath, 2010). In reference to a study on hotel housekeepers and mindset (Heath & Heath, 2010), the authors sarcastically mentioned an American ‘cultural definition of exercise as something we do on a treadmill in a fitness club, while surrounded by spandexed women and perspiring men’. The finding also, unfortunately, suggested that the participants in the study might not be gaining the amount of health benefits than they would if they considered their daily activities as ‘exercise’.

In summary of these additional findings, although not directly related to the research questions, these unexpected research findings have implications for HR development (HRD) and health-promotion professionals. As discussed, a broader understanding of health and well-being in the workplace needs to be generated. Consideration needs to be given toward planning interventions that emphasize all dimensions of health. Additionally, organizational issues need to be addressed in order to foster physical and social work environments that enhance well-being and eliminate factors that detract from well-being.

## Research limitations

Qualitative research provides the flexibility and opportunity to explore phenomena in-depth while allowing the researcher to make decisions throughout the research process from data collection through writing the findings. This very flexibility and opportunity could lead to – or at least cause a perception of – possible researcher bias. The methodology itself is potential for bias related to participant selection and researcher interviewing skills (Bloomberg & Volpe, 2008). Also, the in-depth exploration relied on the participants' perceptions and was limited to only 19 participants; both of which are common to qualitative research. These reasons are the strength and value of qualitative research, and yet are also potential limitations (Bloomberg & Volpe, 2008). Triangulation, peer review, memo-ing, transparency, and participant verification strengthened trustworthiness and validity. Regardless, part of the transparency also means embracing other limitations. This researcher acknowledges that a sample size of 19, while not large, provided volumes of data that would not have been possible to obtain with a survey or to analyze for a larger sample size. Moreover, saturation was reached well before the end of completing the second interview for the participants. New themes were not identified, yet the characteristics became similar – although the stories shared were different and added to the robustness of the findings.

Consequently, the potential limits of biases, sample size, sample characteristics, and methodology were recognized, accepted, and steps were taken to mitigate the potential limitations of the transferability of the findings.

## Summary

These findings supported the importance of the social workplace environment expressed by other authors and researchers, and began to identify the characteristics of the workplace social environment that influences perceptions of health and well-being (DeJoy & Wilson, 2003; Linnan et al., 2001; MacDermid et al., 2008; MacIntosh et.al., 2007; Quick et al., 2007). Future studies can extend this line of inquiry by developing a survey instrument to measure the types and frequencies of enhancing or detracting social interactions as one way toward expanding our knowledge base in this domain. Linking the characteristics with behaviors identified in the incivility research may help one to find effective solutions in creating prosocial working environments, especially interactions between managers toward employees.

Findings suggest implications for the following three practice initiatives.

1. Change the paradigm of HRD and health-promotion behavior change programs to one based on the social ecological framework. Special emphasis needs to include the social environmental aspects of the workplace. This broader focus will equalize the emphasis of health-promotion programs to promote well-being beyond the physical aspects of health. It will thereby expand the repertoire of health promoting activities to include how we interact with others, mindfulness, and other well-being practices.
2. Create interventions that improve interpersonal dynamics, as well as organizational factors supporting the dynamics. This includes establishing a physical and social environment that allows for the pervasiveness of enhancing characteristics and health practices, and eliminates factors that detract from well-being. A collaborative approach between HRD and health-promotion professions can establish best practice for assessing and addressing the social environment.
3. Utilize CoP for implementing sustainable HRD and health-promotion initiatives.

The study offers HRD and health-promotion professionals’ evidence that supports interventions aimed at creating positive work culture environments and minimizing workplace incivility and disrespect. Finding opportunities for partnering with experts in these areas would be extremely beneficial to wellness and health services professionals. In addition, it is ironic that certain factors that determine wellness are not included in many workplace wellness initiatives. The irony has not been lost on employees. Interventions designed to foster leadership, employee engagement, communications, and collegiality should be considered as methods to minimize the financial and human cost of negative social interactions at work. Not doing so may jeopardize the success of an employee wellness program and fuel employee cynicism.

Individual well-being and health practices are inextricably interconnected with organizational well-being. The two should not be viewed independently of one another. For sustainability and success, worksite wellness needs to consider the broader determining factors on health and health behaviors. Allen et al. (2007) acknowledged that where individuals work and how they work have major influences on their lives. Organizations provide economic as well as social resources for employees, and also opportunities of social support for behavior change. The authors' review finds that work satisfaction comes down to fulfillment, purpose, and the social context of the workplace.

## Consider the possibilities

Broader approaches to workplace wellness programs must include cultural aspects to enhance well-being as well as measures to facilitate organizational well-being, such as trust, collaboration, meaningful work, respect, valuing and appreciating all contributions, and developing managers to enhance leadership qualities for each of these characteristics. This is the true benefit of wellness initiatives. The outcomes from organizational and individual well-being can reach far beyond healthcare cost avoidance, such as the influence on employee engagement, retention, attraction, discretionary behaviors, and collaboration. The intention is to interconnect what is needed for individuals to thrive with what an organization needs to thrive. Just as Deci stated, ‘ … ask, not how we can motivate … . but, How can we create the conditions within which others will motivate themselves?’ (Deci, 1995). Picture the possibilities of thriving and flourishing employees at thriving and flourishing organizations:

- Employees looking forward to – and enjoying – coming to work
- Wellness and safety committees that are employee-driven; members are engaged and enabled
- HRD, wellness professionals, and other organizational leaders meet regularly to discuss the social and physical workplace environment to consider sustainable changes needed
- Sustainable wellness options that might include paid volunteer time, work-time to develop ideas for the company, meditation classes and quiet spaces, movement classes, flexibility to exercise, life–work balance initiatives, opportunities to be in nature, ongoing wellness classes in all dimensions of well-being
- Wellness and well-being as components of the culture in making healthy choice easier and more accessible
- Leadership development that embraces mindful awareness and emotional intelligence concepts to foster healthy interpersonal interactions
- Active, ongoing safety emphasis that addresses safety concerns and engages employees in finding solutions
- Groups of employees discussing work while walking on the grounds or taking walking breaks outdoors
- Collaborative teams excitedly working on projects
- Employees comfortable to speak out about issues and concerns without fear of reprisal and finding the support to address solutions
- Walking meetings regularly held by most departments
- Managers and employees working collaboratively, upholding respect for all parties and listening to all opinions
- Collaborative confrontations are welcomed and encouraged. Collaboration is not mere agreement to maintain harmony, but parties are comfortable expressing dissenting views
- Employees engaged in all aspects of the business and enabled to make necessary decisions
- Healthy options in vending areas and cafeterias that are subsidized and prominent with less healthier choices in the background
- Healthy options at meetings when food is served.

The bottom line is that a workplace should be physically, socially, emotionally, and psychologically safe in order for well-being to flourish. The social environment has a significant influence on employee sense of well-being and health behaviors, which reinforces the limitation of wellness initiatives that are only focused on physical health and individual behavior change. Multilevel, multi-professional, and multidimensional approaches are essential for sustainable organization and individual well-being – both of which are inextricably connected.
